# Self-rated health and quality of life among Syrian refugees in Ireland – data from a cross-sectional study

**DOI:** 10.1186/s12889-022-13610-1

**Published:** 2022-06-16

**Authors:** Claire Collins, Ivana Pericin, James Larkin, Esperanza Diaz

**Affiliations:** 1 Irish College of General Practitioners, 4-5 Lincoln Place, Dublin 2, Ireland; 2 Trinity College Dublin, Dublin, Ireland; 3 Royal College of Surgeons, Dublin, Ireland; 4 University of Bergen, Bergen, Norway; 5 Norwegian Institute of Public Health, Oslo, Norway

**Keywords:** Syrian, Refugees, Health status, Quality of life, Self-reported health, Mental health, Chronic pain

## Abstract

**Introduction:**

As a response to the humanitarian crisis in Syria, the Irish government agreed to accept up to 4000 refugees for resettlement in Ireland in 2016. Prior to their arrival in Ireland, health screening was carried out by the International Organisation for Migration. However, no population-level measurement of the health status or needs takes place in Ireland to inform policy or health services requirements.

**Methods:**

Cross-sectional data from a self-completed questionnaire among 194 Syrian Refugees aged 16 years and older resident in reception centres in Ireland in 2017/2018 is reported upon. The questionnaire measured self-reported health including quality of life and all study material were available in English and Arabic. The data was examined applying descriptive statistics and regression analysis.

**Results:**

Syrian Refugees in Ireland consist of a relatively young cohort; in this study the majority of participants were younger than 35 years (69.5%). Two-thirds of the respondents reported their overall health status to be good or very good. The most common health condition was found to be headache and the most common medications used were painkillers. Chronic pain was experienced by one quarter of respondents; 27.5% were considered as suffering from anxiety and 10.0% had symptoms compatible with post-traumatic stress disorder (PTSD). A significant relationship was observed between chronic pain and self-rated health, as well as between chronic pain and anxiety. Quality of life (QoL) scores were lowest for the QoL environment domain.

**Conclusions:**

Chronic pain is relatively widespread among these young and otherwise healthy refugees. Psychological distress and trauma are important factors in respondents’ quality of life scores. Chronic pain is associated with one’s mental health. Our findings and the literature suggests that the diagnosis and treatment of pain and providing care in a culturally sensitive manner should be a priority and included in the preparation and training of the relevant care providers. Additionally, the impact of living conditions on quality of life should not be underestimated.

**Supplementary Information:**

The online version contains supplementary material available at 10.1186/s12889-022-13610-1.

## Introduction

Conflict in Syria initiated one of the largest humanitarian crises to date, causing external and internal displacement of millions of people [1, 2]. As a result of the displacements, more than one million refugees sought asylum in the European Union (EU) [2]. In order to address the refugee crisis and assist the EU states who are most exposed to refugee flows, the majority of the EU member states agreed to take part in a resettlement scheme [2, 3]. As a part of the EU resettlement scheme, and under the Irish Refugee Protection Programme, Ireland committed in 2015 to accepting 4000 refugees [4].

Prior to their arrival in Ireland, refugees commonly faced precarious living conditions, including being accommodated in unsafe, overcrowded spaces, often with the absence of basic hygiene requirements [5]. A lack of access to education, social assistance, protection and medical care have been reported [5]. Previous studies have highlighted that the health issues experienced by refugees are largely heterogeneous, and include a variety of somatic and mental health conditions [6, 7]. At the time of the arrival in the destination country, physical health problems such as respiratory tract infections, rheumatological symptoms, headache/neurological conditions, dermatological diseases, and chronic pain [8, 9] have been most commonly identified within the refugee population. Mental health conditions were found to differ greatly in comparison with host populations, and increased prevalence of depression, anxiety, and post-traumatic stress disorder (PTSD) among newly arrived refugee populations have been reported [10–12]. Some of the previous research suggested an association between mental health symptoms and chronic pain [13–15], and in the case of refugees this could be due to a connection between the nature of traumatic events and physical injuries inflicted as a result of war and torture [16–18]. The provision of appropriate health care is essential on refugee arrival, and during further integration in the host communities. However, refugees have reported unmet health needs at arrival to their destination countries [19–21]. Refugee dissatisfaction was mostly due to long waiting times to be seen by a doctor, costs associated with health service provision, and lack of time to seek health care services [19–21].

At the time of the study, the policy in Ireland advocated that newly arrived Syrian refugees be located in temporary accommodation centres known as Emergency Response and Orientation Centres (EROCs) [22]. The centres aimed to provide a stable, supportive and safe environment for refugees, prior to their housing in permanent homes across Ireland including basic needs along with a range of services such as medical care, language training, education, cultural orientation and social protection services for all the residents [22]. However, direct provision centres generally have been criticised for their poor living conditions [23]. Following the initial health screening provided on arrival, refugees have the same access to healthcare services as Irish citizens under the public healthcare system, with an additional benefit of not having to pay prescription fees. However, although the EROCs provided access to primary care services, a general overview of refugee health remained unavailable.

In order to provide a better quality of life for refuges, and adequate health care, appropriate allocation of resources is required. It is therefore necessary for policy makers and healthcare service planners to understand the healthcare needs of this group. In this article we aim to 1) describe the health status and use of medication among Syrian refugees in Ireland and 2) identify the key factors associated with self-reported adverse health status and quality of life (QoL), showing the relevance for health and social service providers and planning internationally.

## Methods

### Aim, design, and setting of the study

A descriptive, cross-sectional study was undertaken, with the overall aim to investigate self-reported health among newly arrived Syrian refugees in Ireland following the appropriate authorisations from the Department of Justice and the management of each of the four EROC sites. All Syrian refugees arriving in Ireland were housed initially in EROCs. At the time of the data collection, a total of 412 individuals aged 16 years and older were resident in the centres. A population based approach was adopted with those resident in EROCs at the time of the study taken to represent all Syrian refugees in Ireland. No sampling was undertaken with all residents in the EROCs invited to participate in the survey. Data collection in each EROC occurred over one or two consecutive days. In three of the EROCS, residents were housed in hotel type accommodation and in these, data collection occurred in a large room onsite. In the fourth site, residents lived in individual units and for these, the questionnaire was left with the potential respondent and collected later in the day.

#### The characteristics of participants & description of materials

Data were collected by means of a self-completed questionnaire, administered to Syrian refugees aged 16 years and older who were living in EROCs. Participants under the age of 18 years required parental consent to participate. Prior to the commencement of the study, ethical approval was obtained from the Irish College of General Practitioners Research Ethics Committee.

Data collection took place between October 2017 and July 2018 in four EROCs with data collection in three EROCs occurring over 1 day and in the fourth over a two-day period. The paper-based questionnaire collected data on a range of areas including sociodemographic and migration information, health status and health related quality of life (see Additional file 1). The questionnaire was cross culturally validated in Norway utilising the same questionnaire following permissions to use the validated instruments [24]. During questionnaire administration, translators were present in order to assist those with low literacy levels and all survey material was available in both English and Arabic. On the request of centre residents, a presentation was provided on dental health on the day of survey administration although completion of the questionnaire was not linked to attendance. Additionally, an incentive of personal care items was offered to those who completed the questionnaire. The status of refugee was defined as admitted to Ireland under the Irish Refugee Protection Programme [4].

### Survey instrument

The questionnaire used was utilised elsewhere in a similar population [24]. Questions not already validated, such as demographic questions, ever experience of health conditions, medications used, unmet need and migration related questions went through a translation process based on the ISPOR principles of good practice guidelines [20, 24, 25] (See Additional file 1 for questionnaire).

Chronic pain was assessed by a validated question from the International Association for the Study of Pain, which defines pain lasting for more than 6 months as chronic pain [26]. The Hopkins Symptom Checklist (HSCL-10) [27] and the Harvard trauma questionnaire (HTQ) [28] were included in the overall questionnaire to screen for psychological distress and symptoms of post-traumatic stress disorder (PTSD). For each of these questionnaires, at least half of the questions must be answered for the response to be considered valid. These instruments have satisfactory psychometric properties in Arabic-speaking populations [29, 30]. HSCL-10 rates the extent to which symptoms of anxiety and depression have distressed the respondent during the last week on a 4-point Likert scale. We used a mean item score of 1.85 as the threshold for psychological distress (range 1–4), predicting a clinically relevant anxiety or depression [24]. HTQ rates the burden of post-traumatic stress symptoms using the same response scale as HSCL-10. We used a mean item score of 2.5 as the cut-off for symptoms compatible with post-traumatic stress disorder (PTSD) (range 1–4) [24]. The Single General Trauma Item was used to measure exposure to traumatic events relating to the experience of forced migration [31].

As a proxy for general health, self-rated health (SRH) was assessed using a single-item question: How do you consider your health at the moment? This question was answered using a five-point response scale from very poor to very good. The item was dichotomized into a binary measure distinguishing between Good and Very Good compared with Very poor, Poor and Neither. The SRH measure has shown reliability and validity among Arabic speakers and within refugee populations [32, 33].

Quality of Life (QoL) was measured using the WHO Quality of Life Scale (WHOQOL-BREF), which was developed as a transcultural instrument and has demonstrated good psychometric properties, reliability, and validity among Arabic speakers [34]. This QoL instrument comprises 24 items measuring four domains: physical health (seven items), psychological health (six items), social relationships (three items) and environment (eight items). Each item is rated on a 5-point Likert scale with a higher score denoting a better QoL. Raw scores were transformed creating domain scores within the range of 4–20 by multiplying the average of the items in each domain by four, in line with instructions from the instrument manual [35]. Cronbach’s alpha for the total scale for the present sample was 0.8.

Perceived social support was measured with the ENRICHD Social Support instrument (ESSI), a short validated self-reported measure that assesses social support using seven items [36]. A total score is the sum of all items with higher scores indicating better social support. Using an approach applied by others, we created a binary measure for high social support defined as having answered > 2 on at least two of the seven items and a total score of > 18 [20, 36].

The final part of the questionnaire evaluated the access to healthcare, unmet health needs and knowledge of the healthcare system by applying multichoice answers in relevant domains. An insight into unmet health needs was gained using a single-item question ‘If you have experienced unmet health needs mentioned in previous questions, where were you residing?’

### Statistical analysis

Data analyses was carried out using SPSSv28. Descriptive analysis was undertaken. We performed logistic regression to study the relationship of factors to self-rated health. The explanatory variables were chosen based on models from similar analyses [24, 25]. As the scores for the domains of QoL are mainly skewed distributions, non-parametric tests between self-reported health categories were performed using the Kruskal-Wallis test.

A *p*-value of < 0.05 was considered significant.

## Results

In total, 194 questionnaires were completed, representing a 47.1% response rate. The majority (95.3%) of respondents stated that they were from Syria; in the age range 16–34 (69.6%) and married (71.9%). Almost all (93.3%) of married respondents were living with their partners. Overall, 12.1% of respondents arrived into Ireland alone and 93.5% of all respondents had a residents’ permit (Table 1). Overall, 74.4% of all respondents had children ranging from one to five children with a median of three. The number of years spent in education ranged from zero to 19 years with a median of 9 years. Almost all (91.4%) respondents had stayed in a transit country on their way to Ireland and for 32.0% of these this phase lasted more than 2 years. Approximately one fifth (21%) reported being detained against their will in a country prior to arrival in Ireland.

**Table 1:** Demographics and transit related information of respondents

	%	N
Gender (*n* = 192)		
Female	41.1	79
Male	58.9	113
Age group (*n* = 151)		
≤ 24	30.4	46
25–34	39.1	59
35–44	17.9	27
≥ 45	12.6	19
Country of birth (*n* = 190)		
Syria	95.3	182
Iraq	4.2	8
Marital status (*n* = 190)		
Single	22.1	42
Married	72.6	138
Divorced/Separated	2.1	4
Widowed	3.2	6
Occupational status in the country of origin (*n* = 182)		
Employed for wages	12.6	23
Self-employed	23.6	43
Out of work	4.9	9
Homemaker	23.6	43
Student	24.2	44
Unable to work	1.1	2
Other	9.8	18
Resident Permit (*n* = 186)		
Yes	93.5	174
No	6.5	12
If married, are you currently living with your partner (*n* = 134)		
Yes	93.3	125
No	6.7	9
Family arrival status (n = 190)		
Arrived with all family members	55.8	106
Arrived with some family members	32.1	61
Arrived alone	12.1	23
Stayed in a transit country (countries) on way to Ireland (*n* = 185)		
Yes	91.4	169
No	8.6	16
Retained against one’s will during the transit phase (*n* = 181)		
Yes	21.0	38
No	79.0	143

Regarding overall health status, 67.4% of refugees reported their health was either ‘very good’ or ‘good’ at present. The vast majority of refugees (65.3%) did not have any of the conditions listed on the questionnaire. The most common health conditions amongst respondents were arthritis/other joint disease (17.0%), headache (10.3%), and mental health problems (8.2%) (Table 2). Overall, 25.1% reported physical pain which lasted for more than 6 months (chronic pain).

**Table 2:** Percentages of the total population with self-reported health conditions (*n* = 194)

	%	(n)
Arthritis/other joint disease	17.0	(33)
Headache	10.3	(20)
Mental health problems	8.2	(16)
Allergies	7.3	(14)
Abdominal pain/diarrhoea	6.3	(10)
Diabetes	4.6	(9)
Kidney disease	4.1	(8)
Heart related condition	3.6	(7)
Eczema	3.1	(6)
Chronic bronchitis, emphysema, Asthma or COPD	3.1	(6)
Osteoporosis	2.1	(4)
Asthma	1.0	(2)

In terms of medication, painkillers were found to be consumed most on a weekly or daily basis (27.0%) where 13.8% of all respondents used prescribed painkillers and 13.1% used painkillers without prescription.

Participants most commonly expressed that they had ‘quite a bit’ or ‘extreme’ difficulty falling asleep (25.2%), ‘feeling blue’ (25.0%) and ‘feeling hopeless about the future’ (20.4%) in the week prior to completing the questionnaires. Of participants who expressed that they feel ‘extremely’ or ‘quite a bit’ hopeless about the future, eight (26.7%) reported having mental health problems and three (10.0%) reported taking anti-depressive medication daily/weekly.

Amongst those who responded to the relevant questions, 27.5% suffered from anxiety and 10.0% had symptoms compatible with PTSD. The median social support score was 22 (IQR: 13–27) and 75.0% had high social support based on the definition employed. In terms of the four domains of the QoL WHOQOL-BREF instrument, the highest mean ratings were observed for the social relationships domain and the lowest for the environment domain (Table 3).

**Table 3:** Summary of the WHOQOL-BREF domains

Quality of life domains	n	Mean	Median	IQR
Physical health	164	14.4	15.1	12.6, 16.6
Psychological health	166	13.5	14.0	12.0, 16.7
Social relationships	175	14.7	16.0	13.6, 16.0
Environment	172	12.1	12.3	10.0, 14.0

**Fig. 1:**
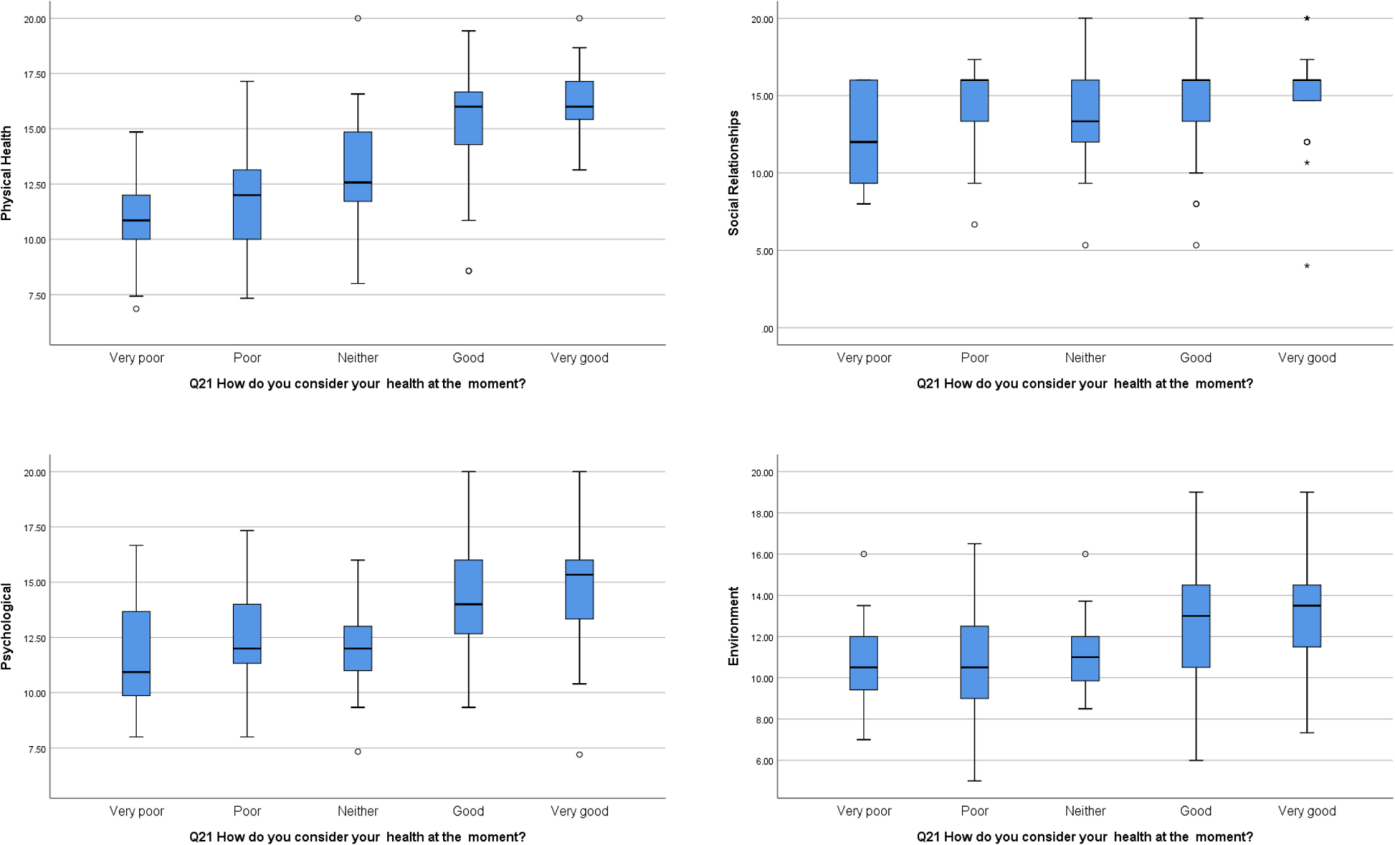
Box Plots of QoL domains and self-rated health

Significant differences were observed between self-rated health responses and the physical health domain scores, the psychological domain scores and the environment domain scores. Those rating their health as good or very good had significantly higher QoL scores in these domains compared to other groups (Fig. 1).

In total, 42.8% had experienced unmet health needs after they flew from Syria. Of these respondents, 53.1% experienced unmet health needs in a transit country, 27.4% in Ireland and 19.4% in both transit country and in Ireland. More than a third of the participants who have experienced unmet health needs (35.7%) considered their health to be ‘poor’ or ‘very poor’, with mental health problems (21.0%), headache (16.1%), and other joint diseases (16.1%) highlighted as the most common conditions. The refugees who experienced unmet health needs in Ireland stated that the most common reason behind this were restrictions/limitations of rights to medical care.

A number of factors were associated with a self-rating of good health (Table 4) in the univariate logistic regression. All significant explanatory variables at the univariate stage were included in the multiple logistical analysis and in this, the only significant variable which remained associated with whether one rated their health as good/very good or not was chronic pain. In this model, those who experience chronic pain were significantly (*p* < 0.01) less likely (OR 0.035 (95%CI: 0.01, 0.13) to rate their health as good.

**Table 4:** Simple univariate logistic regression on factors associated with self-reported good health

	OR (95% CI)	*p*-value
Age	0.96 (0.93, 0.99)	0.006*
Gender (male)	2.42 (1.28, 4.54)	0.026*
Resident Permit	1.15 (0.32, 4.11)	0.826
Unmet Need	0.23 (0.11, 0.49)	< 0.001*
High Social Support	2.69 (1.21, 5.98)	0.015*
Anxiety	0.24 (0.13, 0.42)	< 0.001*
HTQ Average Score	0.40 (0.24, 0.66)	< 0.001*
Chronic Pain	0.57 (0.02, 0.14)	< 0.001*^+^

*Significant association *p* < 0.05. ^+^In a multiple regression with all significant explanatory variables at the univariate stage entered into the model, only chronic pain remained significant

A statistically significant relationship (*p* < 0.01) exists between chronic pain and anxiety with 18.4% of those with no chronic pain reaching the threshold for anxiety using the HSCL-10 compared to 52.6% of those experiencing chronic pain.

## Discussion

The overall health status was reported to be good or very good by two thirds of survey respondents. Only a small number of refugees experienced the health conditions listed on the questionnaire. The most common health condition was headache and the most common medications used were painkillers. Chronic pain was experienced by one quarter of respondents; 27.5% were considered as suffering from anxiety and 10.0% had symptoms compatible with PTSD.

Syrian Refugees in Ireland consist of a relatively young cohort, where the majority of participants were younger than 35 years and male (69.5%). These results corroborate the findings of previous studies where the Syrian population has been described as having a ‘young age structure’ with 58% of the population younger than 24 [37]. In a study of a random sample of 352 Syrian refugees based in a ‘tent-city’ in Turkey, the average age of respondents was 37.6 years, and the gender breakdown was approximately 50–50 [38]. Furthermore, our sample was representative of all Syrians in EROCs in Ireland at the time of data collection with 59.3% of the population in being male and 72.7% aged < 35 years [39] compared to 58.9 and 72.7% respectively in our responding sample.

Comparison to data from Norway using the same questionnaire indicates that the proportion of respondents who self-reported any health conditions, including chronic pain was lower in Ireland [25], while the proportion reporting daily/weekly use of painkiller medication corresponded with the Norwegian study [25]. The high levels of musculoskeletal conditions observed here have been reported elsewhere among similar populations [25, 40]. The average age of the responding sample in this and the Norwegian study, using the same questionnaire and methodology, was similar (mean age 30 years in Ireland compared to 31 years in Norway [25]). However, there were differences related to other demographics, specifically gender (41% female Ireland, 27% female Norway [25]), having children (74% Ireland, 46% Norway [25]), and the percentage who travelled alone (12% Ireland, 49% Norway [25]). These differences should be considered when interpreting the comparative results and could explain the observed differences.

According to the present study, 67.4% of Syrian refugees in Ireland described their health to be either ‘very good’ or ‘good’ at the time of the study. The results from the other research, investigating self-reported health among newly arrived refugees, suggest variability in this regard, ranging from 58% in Norway to more than 70% in Germany and Austria [24, 41]. However, the comparison with the overall health of the Irish population, where 83% of adults reported good overall health [42], indicates a health disparity between the refugee population and Irish nationals, even though the refugees had a younger age profile. Often a variety of factors associated with refugee status, including linguistic, cultural, and financial barriers, provide a plausible explanation for lower health status and inequalities in health provision [43]. The results are indicative of a relationship between chronic pain and underlying mental health problems, where the participants who confirmed chronic pain are also more likely to experience a high level of anxiety. This finding corresponds with the Norwegian study [13], as well as the previous research focused on refugee population [14–17], where it was found that mental health problems, including anxiety, play a significant role in experiencing chronic pain. The strong connection between anxiety and chronic pain could be attributable to exposure to multiple traumas, prior to and post resettlement, and consequently contributes to the restrictive use of adaptive strategies once resettled.

Previous research suggested that the percentage of refugees suffering from anxiety ranged from 26% to as high as 61% [24, 25, 44, 45]. Since the anxiety levels found in this study are on the lower scale (27.5%), there appears to be lower levels of psychological distress in the sample of newly arrived Syrian refugees in Ireland relative to other groups of Syrian refugees. However, this is high when compared with the Irish population where it is reported that 19% of adults experience mental health problems [46]. High levels of anxiety among the refugee population are often associated with increased vulnerabilities to external factors beyond their initial exposure to traumatic events, including difficult living conditions and limited opportunities for development. Inadequate living conditions have been reported in direct provision centres [23] and our results point to lower scores in the environmental domain of QoL.

Previous reports have stressed that the health needs of newly arrived refugees are often found to be incomplete in the host countries, suggesting that between 30 and 61% [19–21] of refugees stated that their health needs were not met. Although a lower percentage in comparison with other studies, 28% of the participants of this study reported the same. Such findings demonstrate a need for further effort in the provision of additional health care for this vulnerable group across multiple countries.

Our results reflect one point in time in the trajectories of these refugees. The literature has pointed to key factors to consider in their interpretation including that self-selection into migration of healthy individuals from a country may occur (referred to as the ‘healthy migrant hypothesis’ [47]), although it is unclear if this exists for forcibly displaced migrants such as refugees. Improvements in health in the early migration phase have also been documented due to factors such as increased safety and hope for the future [48] – the participants in this study were considered newly arrived. However, the ‘exhausted migrant theory’ cautions that deteriorating health outcomes over time are likely due to a range of factors [49].

### Study strengths and limitations

While the study is population based, the responding sample is self-selecting, which comes with inherent bias. Self-selection bias (also referred to as volunteer bias) may have resulted in either higher or lower self-reporting of illness, health status and quality of life. The ‘healthy migrant hypothesis’ [47] may be a factor explaining our results. The study is cross sectional in design; however, it is the first in Ireland to provide a comprehensive overview of the self-reported health of Syrian refugees and it provides data on self-reported lifetime prevalence. Given the potential volunteer bias and the cross-sectional survey design, direct assessment of causal relationships was not possible. The use of self-reported data is subject to recall bias. While our response rate of 47.1% is reasonable, our sample size is relatively small and we acknowledge that this impacts on both the precision and power of our study. A key strength of this study is the use of validated instruments and that the entire questionnaire has also been used elsewhere providing valuable and direct international comparison.

### Implications for policy and practice

The existence of health-related differences in need and mortality between immigrants and their host populations have been previously documented [50–53]. These differences may be in either direction and depend on a variety of factors [54–56]. While most of our respondents self-report good health and self-reported mental illness was low, symptoms such as ‘difficulty falling asleep’, ‘feeling hopeless about future’ and a ‘sudden emotional or physical reaction when reminded of the most hurtful or traumatic events’ were experienced by almost one third of respondents. There is also a relatively high prevalence of headache and use of painkillers. Chronic pain and mental health symptoms are common among refugees [13–17, 57–60] and inadequate healthcare can lead to disimprovement [57]. Untreated symptoms may impact not only on health but also one’s social integration and acculturation [50, 51, 53]. Our public health policies should acknowledge migration related risk and target chronic pain and mental health symptoms [25].

The health of refugees is dynamic and data from longitudinal work has suggested that both short-term and long-term health outcomes should be considered [24]; a critical component is continuity of care [24, 40, 61].

Measures to ensure the provision of culturally sensitive [61–63] and equitable [61, 62] healthcare should be a priority for health policy makers and health service providers. We need to learn from elsewhere in terms of ensuring that population wide and targeted interventions are adapted to be sensitive to diversity and equally effective across society [62–64].

Low quality of life scores and high levels of anxiety among the refugee population have been shown to be associated with environmental factors such as living conditions [10–12] and are a key factor to consider for policy makers and providers in countries accepting refugees.

## Conclusion

The impact of living conditions on quality of life should not be underestimated. Our results also point to the importance of factors such as chronic pain and psychological/emotional distress on respondents’ self-reported health and on quality of life scores. Inadequate healthcare can lead to worsening of symptoms and may impact refugees’ ability to integrate in addition to their health. Measures to ensure culturally sensitive and equitable healthcare should be a priority and health and social care providers should be adequately prepared for caring for refugee populations. The impact of one’s environment on quality of life and self-perceived health is an important consideration across countries accepting refugees.

## Supplementary Information

**Additional file 1.** Questionnaire.

## Data Availability

Anonymous data are available on reasonable request from the corresponding author.
