# Supplementary material for: Self-rated health and quality of life among Syrian refugees in Ireland – data from a cross-sectional study
Source: BMC Public Health. 2022 Jun 16;22:1202. doi: 10.1186/s12889-022-13610-1 (PMC9202096; doi:10.1186/s12889-022-13610-1)
Supplement: Supplementary file 1 — Additional file 1. Questionnaire. [file 12889_2022_13610_MOESM1_ESM.pdf]

Date:

Place:

CHANGING HEALTH AND HEALTH CARE NEEDS ALONG THE SYRIAN REFUGEES' TRAJECTORIES TO IRELAND

QUESTIONNAIRE

Thank you for taking part in this study by completing this questionnaire.

The information will be used in research aimed to understand the health situation and improve health care services for refugees. Some of the questions are similar to questions you answer when you attend the health examination. It is important that you answer all the questions on this questionnaire. Please ask if there is something you do not understand. The completed questionnaire should be returned to the person who invited you to the study before you leave.

By answering this questionnaire you accept that we use this information only for the purposes explained to you. All information will be treated in strict confidence.

This survey contains 5 parts. Please answer by putting an X in the box (☐), or answering the open fields ( ) as explained in the text.

Yours sincerely,
The Irish College of General Practitioners and the Partnership for Health Equity

HEALTH LITERACY SCREENING

1 **How often do you need help reading written material from your doctor or pharmacy?**

Never	Rarely	Sometimes	Often	Always
☐	☐	☐	☐	☐

BACKGROUND INFORMATION

2 **Gender:** ☐ Woman ☐ Man

3 **Year of birth:** ____ *(e.g. 1978)*

4 **Which country were you born in?**

☐ Syria ☐ Iraq ☐ Other

Please specify (e.g. Turkey).

5 **What language is your native tongue?**

☐ Arabic ☐ Kurmanji ☐ Sorani
☐ Armenian ☐ Other

Please specify (e.g. Turkish).

6 **What is your ethnicity?**

☐ Arab ☐ Kurd ☐ Armenian ☐ Other

Please specify (e.g. Turkish).

7 **What is your marital status?**

☐ Single ☐ Separated ☐ Married
☐ Divorced ☐ Widowed ☐ Other

8 **If married, are you living with your partner(s)?** ☐ Yes ☐ No

9 **Do you have children?** ☐ Yes ☐ No

10 **How many children do you have?**

☐ 1 ☐ 2 ☐ 3 ☐ 4 ☐ 5 or more

11 **How many years of education have you completed altogether?** ____ years *(e.g. 5 years)*

Survey number/ID:

12 What was your occupational status in your country of origin?

- ☐ Employed for wages
- ☐ Self-employed
- ☐ Out of work
- ☐ Homemaker
- ☐ Student
- ☐ In the military
- ☐ Retired
- ☐ Unable to work
- ☐ Other

Please explain.

13 When did you flee from your home country?

Year: ____ *(e.g. 2013)*

14 When did you arrive to the country where you are now?

Month and year: ____

(e.g. November 2013)

15 Did you arrive?

- ☐ With all immediate family members
- ☐ With some immediate family members
- ☐ Alone

	Yes	No
**16 Have you stayed in any country (transit) on your way to this place?**	☐	☐

17 If yes, in how many countries did you stay for more than a week?

☐ One ☐ Two ☐ Three ☐ More than three

18 If you have stayed in several countries on the way to this place, for how long (in total) did you stay in that country/ those countries?

- ☐ Up to 6 months
- ☐ 6-12 months
- ☐ 1 -2 years
- ☐ More than two years

	Yes	No
**19 Were you ever retained against your will during the transit phase?**	☐	☐

	Yes	No
**20 Do you have a residence permit in the country you are now?**	☐	☐

Survey number/ID:

HEALTH STATUS

21 How do you consider your health at the moment?

Very poor	Poor	Neither	Good	Very good
☐	☐	☐	☐	☐

22 Have you had or do you have any of the following?
(Put an X on each line under No or Yes. If Yes, please explain.)

	Not familiar with the term	No	Yes	Age first time
22.1 Heart attack/chest pain	☐	☐	☐	22.2 ___ *years*
22.3 Heart failure	☐	☐	☐	22.4 ___ *years*
22.5 Other heart disease	☐	☐	☐	22.6 ___ *years*
22.7 Stroke/brain hemorrhage	☐	☐	☐	22.8 ___ *years*
22.9 Kidney disease	☐	☐	☐	22.10 ___ *years*
22.11 Liver disease	☐	☐	☐	22.12 ___ *years*
22.13 Asthma	☐	☐	☐	22.14 ___ *years*
22.15 Chronic bronchitis, emphysema or COPD	☐	☐	☐	22.16 ___ *years*
22.17 Tuberculosis	☐	☐	☐	22.18 ___ *years*
22.19 Diabetes	☐	☐	☐	22.20 ___ *years*
22.21 Psoriasis	☐	☐	☐	22.22 ___ *years*
22.23 Eczema	☐	☐	☐	22.24 ___ *years*
22.25 Cancer	☐	☐	☐	22.26 ___ *years*
22.27 Arthritis *Reumatoid arthritis*	☐	☐	☐	22.28 ___ *years*
22.29 Other joint diseases	☐	☐	☐	22.30 ___ *years*
22.31 Osteoporosis	☐	☐	☐	22.32 ___ *years*
22.33 Fibromyalgia or generalized body pain	☐	☐	☐	22.34 ___ *years*
22.35 Mental health problems you sought help for	☐	☐	☐	22.36 ___ *years*
22.37 Epilepsy	☐	☐	☐	22.38 ___ *years*
22.39 Headache	☐	☐	☐	22.40 ___ *years*
22.41 Abdominal pain/diarrhea	☐	☐	☐	22.42 ___ *years*
22.43 Allergies	☐	☐	☐	22.44 ___ *years*

23 Do you suffer from long-term (at least 1 year) illness or injury of a physical or psychological nature that impairs your daily life?

☐ Yes ☐ No

24 If yes, how would you describe your impairment?

	Slight	Moderate	Severe
24.1 Motor ability impairment	☐	☐	☐
24.2 Vision impairment	☐	☐	☐
24.3 Hearing impairment	☐	☐	☐
24.4 Impairment due to physical illness	☐	☐	☐
24.5 Impairment due to mental health problems	☐	☐	☐

	Yes	No
**25 Do you have physical pain now that has lasted more than 6 months?**	☐	☐

26 If yes, how strong has your physical pain been during the last 4 weeks?

No pain	Very mild	Mild	Moderate	Strong	Very strong
☐	☐	☐	☐	☐	☐

27 Have you used any of the following medicines?
(Please place only one X for each medication at the answer that best fits your situation.)

	Daily	Weekly	Less than weekly	Not taken during the last 4 weeks
27.1 Drugs for peptic ulcer, gastro-esophageal reflux and digestion	☐	☐	☐	☐
27.2 Antithrombotics (aspirin, warfarin)	☐	☐	☐	☐
27.3 Cholesterol reducing medication	☐	☐	☐	☐
27.4 Medicine for high blood pressure	☐	☐	☐	☐
27.5 Medicine for diabetes mellitus	☐	☐	☐	☐
27.6 Medication for asthma or COPD	☐	☐	☐	☐
27.7 Painkillers, off prescription	☐	☐	☐	☐
27.8 Painkillers, on prescription	☐	☐	☐	☐
27.9 Sedatives	☐	☐	☐	☐
27.10 Tranquillizers	☐	☐	☐	☐
27.11 Anti-depressive medication	☐	☐	☐	☐
27.12 Medication for allergy	☐	☐	☐	☐
27.13 Other prescribed medication, but do not know for what	☐	☐	☐	☐

28 Listed below are symptoms or problems people sometimes have. Please indicate in the appropriate box how much each of these symptoms has bothered or distressed you <u>in the last week</u>.

	None	A little	Quite a bit	Extremely
28.1 Suddenly scared for no reason	☐	☐	☐	☐
28.2 Feeling fearful	☐	☐	☐	☐
28.3 Faintness, dizziness or weakness	☐	☐	☐	☐
28.4 Feeling tense or keyed up	☐	☐	☐	☐
28.5 Blaming yourself for things	☐	☐	☐	☐
28.6 Difficulty falling asleep, staying asleep	☐	☐	☐	☐
28.7 Feeling blue	☐	☐	☐	☐
28.8 Feeling of worthlessness	☐	☐	☐	☐
28.9 Feeling everything is an effort	☐	☐	☐	☐
28.10 Feeling hopeless about future	☐	☐	☐	☐

29 Exposure to a stressful event or situation (either short or long lasting) of exceptionally threatening or catastrophic nature is likely to cause pervasive distress in almost anyone. Examples of such difficult and frightening experiences are: being assaulted, or witnessing other people being hurt or killed.

	Yes	No
Have you experienced any of these or some other terrifying event(s)?	☐	☐

30 The following are symptoms people sometimes experience after hurtful and terrifying events. Please indicate, in the appropriate box, how much each symptom has bothered you <u>in the last week</u>.

	Not at all	A little	Quite a bit	Extremely
30.1 Recurrent thoughts or memories of the most hurtful or terrifying events	☐	☐	☐	☐
30.2 Feeling as though the event is happening again	☐	☐	☐	☐
30.3 Recurrent nightmares	☐	☐	☐	☐
30.4 Feeling detached or withdrawn from people	☐	☐	☐	☐
30.5 Unable to feel emotions	☐	☐	☐	☐
30.6 Feeling jumpy, easily startled	☐	☐	☐	☐
30.7 Difficulty concentrating	☐	☐	☐	☐
30.8 Trouble sleeping	☐	☐	☐	☐
30.9 Feeling on guard	☐	☐	☐	☐
30.10 Feeling irritable or having outbursts of anger	☐	☐	☐	☐

Survey number/ID:

30.11 Avoiding activities that remind you of the traumatic or hurtful event ☐ ☐ ☐ ☐

30.12 Inability to remember parts of the most hurtful or traumatic events ☐ ☐ ☐ ☐

30.13 Less interest in daily activities ☐ ☐ ☐ ☐

30.14 Feeling as if you don't have a future ☐ ☐ ☐ ☐

30.15 Avoiding thoughts or feelings associated with the traumatic or hurtful events ☐ ☐ ☐ ☐

30.16 Sudden emotional or physical reaction when reminded of the most hurtful or traumatic events ☐ ☐ ☐ ☐

HEALTH HABITS

31.1 Do you smoke? ***(Put an X in only one box)***

- ☐ No, I have never smoked.
- ☐ No, I quit smoking.
- ☐ Yes, cigarettes occasionally (parties/vacation, not daily).
- ☐ Yes, cigar/cigarillos/pipe/shisha (water pipe) occasionally.
- ☐ Yes, cigarettes daily. Number of cigarettes per day: ☐ 31.2
- ☐ Yes, cigar/cigarillos/pipe/shisha (water pipe) daily.
  Number per day: ☐ 31.3

32 About how often in the last 12 months did you drink alcohol?
(Put an X in only one box)

- ☐ 4-7 times a week
- ☐ 2-3 times a week
- ☐ About once a week
- ☐ 2-3 times a month
- ☐ About once a month
- ☐ A few times a year
- ☐ None the last year
- ☐ Never drink alcohol

	Yes	No
**33 Did you drink alcohol during the past 4 weeks?**	☐	☐

34 If yes, did you drink so much that you felt very intoxicated (drunk)?

- ☐ Yes, 3 times or more
- ☐ Yes, 1-2 times
- ☐ No

	Yes	No
**35 Did you use any other type of drug during the past 4 weeks?**	☐	☐

36 How often do you exercise?
(On average. Put an X in only one box)

- ☐ Never
- ☐ Less than once a week
- ☐ Once a week
- ☐ 2-3 times a week
- ☐ Nearly every day

37 About how many hours do you sit during a normal day?
(Both work hours and leisure time)

About ☐ hours *(e.g. 6 hours)*

HEALTH RELATED QUALITY OF LIFE

	Very poor	Poor	Neither	Good	Very good
38 How would you rate your quality of life?	☐	☐	☐	☐	☐

	Very dissatisfied	Dissatisfied	Neither satisfied nor dissatisfied	Satisfied	Very satisfied
39 How satisfied are you with your health?	☐	☐	☐	☐	☐

	Not at all	A little	A moderate amount	Very much	An extreme amount
40 To what extent do you feel that physical pain prevents you from doing what you need to do?	☐	☐	☐	☐	☐
41 How much do you need any medical treatment to function in your daily life?	☐	☐	☐	☐	☐
42 How much do you enjoy life?	☐	☐	☐	☐	☐
43 To what extent do you feel your life to be meaningful?	☐	☐	☐	☐	☐

Survey number/ID:

	Not at all	A little	A moderate amount	Very much	Extremely
44 How well are you able to concentrate?	☐	☐	☐	☐	☐
45 How safe do you feel in your daily life?	☐	☐	☐	☐	☐
46 How healthy is your physical environment?	☐	☐	☐	☐	☐

	Not at all	A little	Moderately	Mostly	Completely
47 Do you have enough energy for everyday life?	☐	☐	☐	☐	☐
48 Are you able to accept your bodily appearance?	☐	☐	☐	☐	☐
49 Have you enough money to meet your needs?	☐	☐	☐	☐	☐
50 How available to you is the information that you need in your day-to-day life?	☐	☐	☐	☐	☐
51 To what extent do you have the opportunity for leisure activities?	☐	☐	☐	☐	☐

	Very poor	Poor	Neither	Good	Very good
52 How well are you able to get around?	☐	☐	☐	☐	☐

	Very dissatisfied	Dissatisfied	Neither satisfied nor dissatisfied	Satisfied	Very satisfied
53 How satisfied are you with your sleep?	☐	☐	☐	☐	☐
54 How satisfied are you with your ability to perform your daily living activities?	☐	☐	☐	☐	☐
55 How satisfied are you with your capacity for work?	☐	☐	☐	☐	☐
56 How satisfied are you with yourself?	☐	☐	☐	☐	☐
57 How satisfied are you with your personal relationships?	☐	☐	☐	☐	☐
58 How satisfied are you with your sex life?	☐	☐	☐	☐	☐
59 How satisfied are you with the support you get from your friends?	☐	☐	☐	☐	☐
60 How satisfied are you with the conditions of your living place?	☐	☐	☐	☐	☐
61 How satisfied are you with your access to health services?	☐	☐	☐	☐	☐
62 How satisfied are you with your transport?	☐	☐	☐	☐	☐

	Never	Seldom	Quite often	Very often	Always
63 How often do you have negative feelings such as blue mood, despair, anxiety, depression?	☐	☐	☐	☐	☐

Please read the following questions and put an X for each question in the response that most closely describes your current situation.

	None of the time	A little of the time	Some of the time	Most of the time	All of the time
64 Is there someone available to you whom you can count on to listen to when you need to talk?	☐	☐	☐	☐	☐
65 Is there someone available to give you good advice about a problem?	☐	☐	☐	☐	☐
66 Is there someone available to you who shows you love and affection?	☐	☐	☐	☐	☐
67 Is there someone available to help you with daily chores?	☐	☐	☐	☐	☐
68 Can you count on anyone to provide you with emotional support (talking over problems or helping you make a difficult decision)?	☐	☐	☐	☐	☐
69 Do you have as much contact as you would like with someone you feel close to, someone in whom you can trust and confide?	☐	☐	☐	☐	☐

Survey number/ID:

ACCESS TO HEALTHCARE, UNMET HEALTH NEEDS AND KNOWLEDGE OF HEALTH CARE SYSTEM

70 During the last 12 months, have you visited any of the following:
(Please, put an X on each line)

	Yes	No
70.1 Health assessment at arrival to your current living place	☐	☐
70.2 General practitioner	☐	☐
70.3 Another specialist outside the hospital	☐	☐
70.4 Consultation with doctor without being admitted	☐	☐
70.5 Emergency room services	☐	☐
70.6 Chiropractor	☐	☐
70.7 Homeopath, acupuncturist or other alternative treatment practitioner	☐	☐
70.8 Have you been admitted to hospital in the last 12 months?	☐	☐

71 If you did not get the health care you needed after you fled from your country of origin, was the reason that:
(You may choose more than one option.)

☐ I have not experienced unmet health needs
☐ I did not know where to go for treatment.
☐ Interpreters or cultural mediators were unavailable.
☐ I could not afford it.
☐ The problem was not considered urgent enough.
☐ The services needed were unavailable in my location.
☐ Restrictions/limitations of rights to medical care.
☐ I did not trust the local health services.
☐ Other reasons. Please specify below.

Please specify.

72 Do you feel that in your current living place, you or your family members have access to medical care when you are concerned of your health?

☐ Not at all ☐ A little ☐ Moderately ☐ Completely

73 Do you feel that in your current living place, you or your family members have received the medical assistance you need?

☐ Not at all ☐ A little ☐ Moderately ☐ Completely

74 If you have experienced unmet health needs mentioned in previous questions, where were you residing?

☐ I have not experienced unmet health needs
☐ In a transit country (Lebanon, Greece, Turkey)
☐ In Ireland
☐ Both in transit country and in Ireland

75 Do you currently know where you can find healthcare if needed?

☐ Unsure
☐ No
☐ Yes

THANK YOU FOR ANSWERING THESE QUESTIONS! PLEASE MAKE SURE TO RETURN THIS FORM TO THE PERSON WHO GAVE IT TO YOU BEFORE LEAVING.

Survey number/ID:
